# Patch-testing for the management of hypersensitivity reactions to second-line anti-tuberculosis drugs: a case report

**DOI:** 10.1186/1756-0500-7-537

**Published:** 2014-08-15

**Authors:** Samsuddin Khan, Aristomo Andries, Asha Pherwani, Peter Saranchuk, Petros Isaakidis

**Affiliations:** Médecins Sans Frontières, Chandni Bungalow, Union Park, Off Carter Road, Khar (W), Mumbai, 400 052 India; Allergy Immunology Department, P.D. Hinduja Hospital Research Center, Mumbai, India; Southern Africa Medical Unit (SAMU), Médecins Sans Frontières, Cape Town, South Africa

**Keywords:** HIV, Drug-resistant TB, Co-infection, Drug allergy, Skin patch test

## Abstract

**Background:**

The second-line anti-tuberculosis drugs used in the treatment of multidrug-resistant tuberculosis often cause adverse events, especially in patients co-infected with the human immunodeficiency virus. Severe hypersensitivity reactions due to these drugs are rare and there is little published experience to guide their management.

**Case presentation:**

A 17-year old Indian female multidrug-resistant tuberculosis patient co-infected with human immunodeficiency virus developed a hypersensitivity reaction after starting second-line anti-tuberculosis treatment in Mumbai, India. The patient was being treated with kanamycin, moxifloxacin, para-aminosalicylic acid, cycloserine, clofazimine, and amoxicillin-clavulanic acid. Twenty-four hours later, the patient developed generalized urticaria, morbilliform rash and fever. All drugs were suspended and the patient was hospitalised for acute management. Skin patch-testing was used to identify drugs that potentially caused the hypersensitivity reaction; results showed a strong reaction to clofazimine, moderate reaction to kanamycin and mild reaction to cycloserine. An interim second-line anti-tuberculosis regimen was prescribed; cycloserine and kanamycin were then re-challenged one-by-one using incremental dosing, an approach that allowed clinicians to re-introduce these drugs promptly and safely. The patient is currently doing well.

**Conclusions:**

This is the first case-report of a multidrug-resistant tuberculosis patient co-infected with the human immunodeficiency virus with hypersensitivity reaction to multiple second-line anti-tuberculosis drugs. Skin patch-testing and controlled re-challenge can be a useful management strategy in such patients. There is an urgent need for second-line anti-tuberculosis regimens that are more effective, safe and better tolerated.

## Background

The management of patients with multidrug-resistant tuberculosis (MDR-TB), defined as resistance to isoniazid and rifampicin, is onerous, particularly in those co-infected with the human immunodeficiency virus (HIV) [1, 2]. The second-line anti-TB drugs used in MDR-TB treatment regimens frequently cause adverse events (AE) [3]. Severe hypersensitivity reactions such as anaphylaxis are rare but do occur with these drugs [4]. In case of a severe AE, the culprit drug must be substituted with another drug if available. However, several drugs in the treatment regimen may be responsible for a severe AE, including hypersensitivity reaction, making timely identification of the culprit drug(s) and substitution challenging. Substituting all possible culprit drugs in such cases is not possible due to the limited number of drugs currently available to treat MDR-TB [4].

Skin patch-testing has traditionally been used to identify agents involved in type I and type IV hypersensitivity reactions. Type I hypersensitivity is an immediate immune reaction to an antigen, typically involving mast cells and basophil degranulation, which release inflammatory mediators and cause hives, redness, and angioedema; such symptoms are referred to as anaphylactic reaction. Type IV hypersensitivity is a delayed-type hypersensitivity reaction which can take more than 12 hours to develop. This reaction involves sensitized T memory cells that respond with stronger immune reaction to subsequent exposure with the same antigen. Type IV hypersensitivity is observed in contact dermatitis which is characterized by rash [5].

In this case report we describe our experience with the use of patch-testing to manage hypersensitivity reaction due to multiple drugs in a MDR-TB patient co-infected with HIV being treated with second-line anti-TB drugs and antiretroviral therapy (ART).

## Case presentation

A 17-year old Indian female was diagnosed with HIV infection in January 2011 in a public ART centre. Her baseline CD4 cell count at the time of diagnosis was 428 cells/mm^3^. The patient was not initiated on ART at baseline as the national HIV program in India has a threshold of 350 cells/mm^3^ for ART enrollment in adolescent and adult patients [6]. Co-trimoxazole preventive therapy was initiated when the patient’s CD4 count fell to 340 cells/mm^3^ in February 2013, nevertheless, ART was not initiated at that time.

The patient was diagnosed with pulmonary MDR-TB in June 2013 and referred to the Médecins Sans Frontières (MSF) project. She was treated for pulmonary tuberculosis one year earlier by a private practitioner with first-line anti-TB treatment regimen consisting of isoniazid, rifampicin, pyrazinamide and ethambutol, plus the addition of ofloxacin. The drug susceptibility test (DST) result to this second episode of TB showed resistance of the of the *Mycobacterium tuberculosis* strain to isoniazid, rifampicin, ethambutol, pyrazinamide, streptomycin, ofloxacin and ethionamide. Peripheral full blood count (FBC), including a differential white cell count, creatinine, alanine aminotransferase (ALT), and potassium were performed as baseline tests prior to initiation of drug-resistant TB treatment and found to be normal. The CD4 count at the time of enrollment in the MSF project was 205 cells/mm^3^ and the HIV viral load result was 29,420 copies/ml. The patient’s weight on admission was 40.3 Kg. There was no history of allergic reactions.

After counselling the patient and caregiver, and identifying a health care worker to directly observe therapy (i.e. a DOT provider), an individualized regimen was devised, based on the DST result. The regimen contained capreomycin, moxifloxacin, para-aminosalicylic acid (PAS), cycloserine, amoxicillin/clavulanic acid (amoxiclav) and clofazimine. ART was to be initiated within 14 to 30 days after anti-TB treatment initiation, after the patient was found to be tolerating the anti-TB treatment.

However, twenty-four hours after TB treatment initiation, the patient developed generalised urticarial and morbilliform rash, fever, angioedema, laryngospasm, and dyspnoea. She was immediately referred and admitted to a hospital. All drugs were suspended. During admission, the symptoms and signs of the hypersensitivity reaction subsided. The patient was observed for 48 hours for any late hypersensitivity reaction and then was discharged. The FBC, liver function, and renal function tests did not show any abnormality.

To help identify the drug(s) responsible for the hypersensitivity reaction, a careful re-challenge plan was made. The following 4-step strategy was used to manage the patient’s hypersensitivity reaction related to second-line anti-TB drugs:

### Step 1: Skin patch-testing

The patient was referred to a specialized clinic for skin patch-testing one week following the anaphylactic episode and after antihistamine medication had been stopped. The patch-testing included the following drugs: PAS, moxifloxacin, amoxiclav, cycloserine, kanamycin, clofazimine, and cotrimoxazole. The test showed an extremely positive reaction (+3) to clofazimine, strongly positive reaction (+2) to kanamycin and weakly positive reaction (+1) to cycloserine. No reaction was observed to moxifloxacin, amoxiclav, PAS, and cotrimoxazole. We decided to permanently suspend the use of clofazimine and to re-challenge with cycloserine and kanamycin.

### Step 2: Introduction of an interim MDR-TB treatment regimen

We initiated the patient on an interim MDR-TB treatment regimen consisting of drugs that were less likely to cause hypersensitivity reactions. This regimen included moxifloxacin 400 mg OD, PAS 9.2 gram divided in two doses/day, amoxiclav 625 mg TD, linezolid 600 mg OD and clarithromycin 500 mg BD. The first doses were given under direct observation at the clinic. We observed the patient for 2 hours after administration of the medication and discharged her with no new symptoms or signs. The interim regimen was continued for one week. The patient underwent testing at the end of the week consisting of a FBC, ALT, and serum creatinine. The FBC was unremarkable: hemoglobin (Hb) was 11.4 g/dL, platelets were 150,000 /μL, and the white cell count was 4100 /μL, with a differential of 48% neutrophils, 43% lymphocytes, and 1% eosinophils. ALT was 54.6 U/L and serum creatinine was 0.7 mg/dL (creatinine clearance was 82.98 ml/min).

### Step 3: Re-challenge with drugs having moderate and mild skin-patch test results

The patient was admitted to the hospital for twelve days for re-challenging with cycloserine and kanamycin. Cycloserine was started on Day 1 in escalating dosages as shown in the Table 1.

**Table 1:** **Re-challenge dose of cycloserine and kanamycin**

Prescribed medicine	Day 1	Day 2	Day 3	Day 4	Day 5-7	Remarks
Cycloserine (mg)	12.5 OD	62.5 OD	125 OD	250 OD	250 BD	No reactions
**Prescribed medicine**	**Day 8**	**Day 9**	**Day 10**	**Day 11**	**Day 12**	**Remarks**
Kanamycin (mg)	12.5 OD	62.5 OD	125 OD	300 OD	600 OD	No reactions

OD = once daily; BD = twice daily; TD = three times/day.

Since the patient tolerated both cycloserine and kanamycin, the MDR-TB treatment regimen was then modified as follows: kanamycin 600 mg 6 days per week, moxifloxacin 400 mg (OD), cycloserine 500 mg (OD), PAS 9.2 gram (BD), linezolid 600 mg (OD), and amoxiclav 625 mg (TD).

### Step 4: Introduction of prophylaxis for opportunistic infections and antiretroviral therapy

Two weeks after the MDR-TB treatment regimen was modified and once we confirmed that it was well tolerated, cotrimoxazole prophylaxis was re-initiated to help prevent other opportunistic infections. Two weeks later, we initiated antiretroviral therapy consisting of tenofovir, lamivudine and efavirenz. The patient tolerated the cotrimoxazole and antiretrovirals very well.

The patient’s sputum quickly converted, with the acid fast bacilli (AFB) and culture results becoming negative one month after initiation of the anti-TB regimen. The culture for *M. tuberculosis* remained negative up to the time of reporting this study. Two months after ART initiation, the HIV-1 viral load became undetectable and the CD4 count had increased to 485 cells/mm^3^. The patient was clinically stable, with no medical complaints on the last appointment before reporting of this study, and no symptoms of hypersensitivity reaction.

## Discussion

Hypersensitivity reactions to second-line anti-TB drugs are rare but not completely unknown. There are limited reports available about hypersensitivity to PAS and ethionamide as second-line medicines commonly used in MDR-TB regimen [7–9]. In other studies, the same two second-line anti-TB medicines were associated with hepatotoxicity, while fluoroquinolones and cycloserine were mentioned to have a lower risk for hepatotoxicity [10, 11]. These studies did not describe hypersensitivity reactions associated with other anti-TB medicines that are commonly added in patients with *M. tuberculosis* strains resistant to aminoglycosides and/or fluoroquinolones: clofazimine, linezolid, and amoxicillin-clavulanic acid. Hypersensitivity can manifest from symptoms suc h as rash and pruritus to severe reactions such as Drug Reaction with Eosinophilia and Systemic Symptoms (DRESS), anaphylaxis, Stevens-Johnson Syndrome (SJS) and Toxic Epidermal Necrolysis (TEN) [4].

Patch-testing is a diagnostic procedure that is most commonly used for identifying the possible causes of contact dermatitis, which is a type IV hypersensitivity reaction. Patch-testing can also be used in the investigation of type I hypersensitivity reactions and in DRESS syndrome (type IVb hypersensitivity reaction) [5, 12]. If done properly, the test has good sensitivity, specificity, accuracy and relevance. The procedure involves mixing the allergen to be tested with white petrolatum (i.e. the vehicle) and applied in close proximity with the skin. The first reading is taken after 30 minutes to look for type 1 hypersensitivity reaction, while a second reading is taken after 48 hours to investigate for type 4 delayed hypersensitivity reaction. Between the 2 readings, the patient should be instructed not to wet, rub or scratch the testing area, avoid exercise and sweating. Patch test readings are evaluated using the International Contact Dermatitis Research Group (ICDRG) grading system: ‘-‘stands for negative, ‘+’ stands for a weak (non-vesicular) positive reaction, ‘++’ stands for a strong (vesicular) positive reaction and ‘+++’ stands for extreme (bullous) positive reaction. In general, the accuracy of patch-testing in determining the cause when there is a weakly positive reaction is 20%-50%, compared to 80%-90% with a strong reaction and 95%-100% with an extreme positive reaction. If patch testing is not done properly, there are high chances of false positive and false negative results [13].

In MDR-TB patients co-infected with HIV, early anti-TB treatment initiation with an effective regimen is essential, as pre-treatment mortality among these patients is high [14, 15]. Identifying the drugs least likely to have caused the hypersensitivity reaction is important, as it allows clinicians to design and introduce an individualized regimen that can be started as soon as the person is clinically stable. Use of an interim MDR-TB regimen allows treatment to be continued while the clinician investigates and determines the most likely culprit drug.

In this case, we did not re-challenge the patient with the drug having an extremely strong positive reaction on patch-testing, clofazimine. Instead of re-challenging with clofazimine, we administered linezolid, a group-5 anti –TB drug that was already being given in the interim regimen (2). We opted to re-challenge with only those anti-TB drugs having less strong skin-patch test results, and this was done in ascending order of suspicion, i.e. the drug with the mild reaction (cycloserine) before the one with the stronger reaction (kanamycin).

Introduction of cotrimoxazole prophylaxis and antiretroviral therapy (ART) are important to further reduce the risk of morbidity and mortality in co-infected patients. ART initiation is usually postponed for several weeks after anti-TB treatment to reduce the risk of immune reconstitution inflammatory syndrome (IRIS) [16]. However, ART should not be excessively delayed, as doing so increases the risk of mortality [16].

Guidelines are currently lacking on management of patients experiencing allergic reactions to multiple second-line anti-TB drugs. There are no evidence-based recommendations or expert opinions on how to design an interim regimen and how best to re-challenge possible culprit drugs, including their optimal sequence, dosage and timing schemes. Current guidance tends to be limited to the management of non-anaphylactic allergic reactions [4], management of hepatitis due to first-line (but not second-line) anti-TB drugs or anaphylactic reactions [11], as well as case reports on the management of allergic reactions in patients on first-line anti-TB treatment [17].

## Conclusions

To our knowledge this is the first case report of an HIV/MDR-TB co-infected patient with hypersensitivity reactions to multiple second-line anti-TB drugs. Our experience showed that skin-patch testing and timed re-challenging was a useful part of the management strategy in a MDR-TB patient co-infected with HIV having allergic reactions to multiple second-line anti-TB drugs. By utilizing skin-patch testing, we managed to identify certain anti-TB medicines that were less likely to be the cause of the hypersensitivity reaction and keep them in the post-reaction interim regimen. Similar to the management of hypersensitivity when seen with first-line anti-TB medicines, an interim regimen is important to prevent further resistance against anti-TB medicines [11]. Instead of re-challenging all six anti-TB medicines one-by-one, which could require a total of 42 days, we were able to safely identify anti-TB medicines that did not require re-challenge by utilizing skin-patch testing, and by doing so shortened the time of the re-challenge process by half.

The entire process might have been avoided if a simpler regimen existed to treat MDR-TB. There is an ongoing and urgent need for MDR-TB treatment regimens that are more effective, better tolerated, and consisting of fewer medicines.

## Consent

Written informed consent to publish this case report was obtained from the patient after she had turned 18 years old.
